# DRD2 activation inhibits choroidal neovascularization in patients with Parkinson’s disease and age-related macular degeneration

**DOI:** 10.1172/JCI174199

**Published:** 2024-07-16

**Authors:** Thibaud Mathis, Florian Baudin, Anne-Sophie Mariet, Sébastien Augustin, Marion Bricout, Lauriane Przegralek, Christophe Roubeix, Éric Benzenine, Guillaume Blot, Caroline Nous, Laurent Kodjikian, Martine Mauget-Faÿsse, José-Alain Sahel, Robin Plevin, Christina Zeitz, Cécile Delarasse, Xavier Guillonneau, Catherine Creuzot-Garcher, Catherine Quantin, Stéphane Hunot, Florian Sennlaub

**Affiliations:** 1 Sorbonne Université, INSERM, CNRS, Institut de la Vision, Paris, France.; 2 Hopital de la Croix-Rousse, Hospices Civils de Lyon, Lyon, France.; 3 UMR-CNRS 5510, MATEIS, Institut National des Sciences Appliquées, Université Lyon 1, Campus de la Doua, Villeurbanne, France.; 4 Service d’ophtalmologie, CHU Dijon, Dijon, France.; 5 Ramsaysanté, Clinique d’Argonay, Argonay, France.; 6 Service de Biostatistiques et D’Information Médicale (DIM), CHU Dijon Bourgogne, INSERM, Université de Bourgogne, CIC 1432, Module Épidémiologie Clinique, Dijon, France.; 7 Fondation Ophtalmologique Adolphe de Rothschild, Paris, France.; 8 Department of Ophthalmology, University of Pittsburgh School of Medicine, Pittsburgh, Pennsylvania, USA.; 9 Strathclyde Institute for Pharmacy and Biomedical Sciences, University of Strathclyde, Glasgow, United Kingdom.; 10 Université Paris-Saclay, University of Versailles Saint-Quentin-en-Yvelines (UVSQ), INSERM, Centre for Epidemiology and Population Health (CESP), Villejuif, France.; 11 Sorbonne Université, Paris Brain Institute–L’Institut du Cerveau, INSERM, CNRS, Hôpital de la Pitié Salpêtrière, Paris.

**Keywords:** Neuroscience, Ophthalmology, Neurodegeneration, Parkinson disease, Retinopathy

## Abstract

Neovascular age-related macular degeneration (nAMD) remains a major cause of visual impairment and puts considerable burden on patients and health care systems. l-DOPA–treated Parkinson’s disease (PD) patients have been shown to be partially protected from nAMD, but the mechanism remains unknown. Using murine models that combine 1-methyl-4-phenyl-1,2,3,6-tetrahydropyridine–induced (MPTP-induced) PD and laser-induced nAMD with standard PD treatment of l-DOPA/DOPA-decarboxylase inhibitor or specific dopamine receptor inhibitors, we here demonstrate that l-DOPA treatment–induced increase of dopamine-mediated dopamine receptor D2 (DRD2) signaling inhibits choroidal neovascularization independently of MPTP-associated nigrostriatal pathway lesion. Analyzing a retrospective cohort of more than 200,000 patients with nAMD receiving anti-VEGF treatment from the French nationwide insurance database, we show that DRD2 agonist–treated PD patients have a significantly delayed age of onset of nAMD and reduced need for anti-VEGF therapies, similar to the effects of the l-DOPA treatment. While providing a mechanistic explanation for an intriguing epidemiological observation, our findings suggest that systemic DRD2 agonists might constitute an adjuvant therapy to delay and reduce the need for anti-VEGF therapy in patients with nAMD.

## Abstract 2

Systemic DRD2 agonists, which are generally given for Parkinson’s disease, provide protection against exudative age-related macular degeneration via blocking choroidal neovascularization.

## Graphical abstract

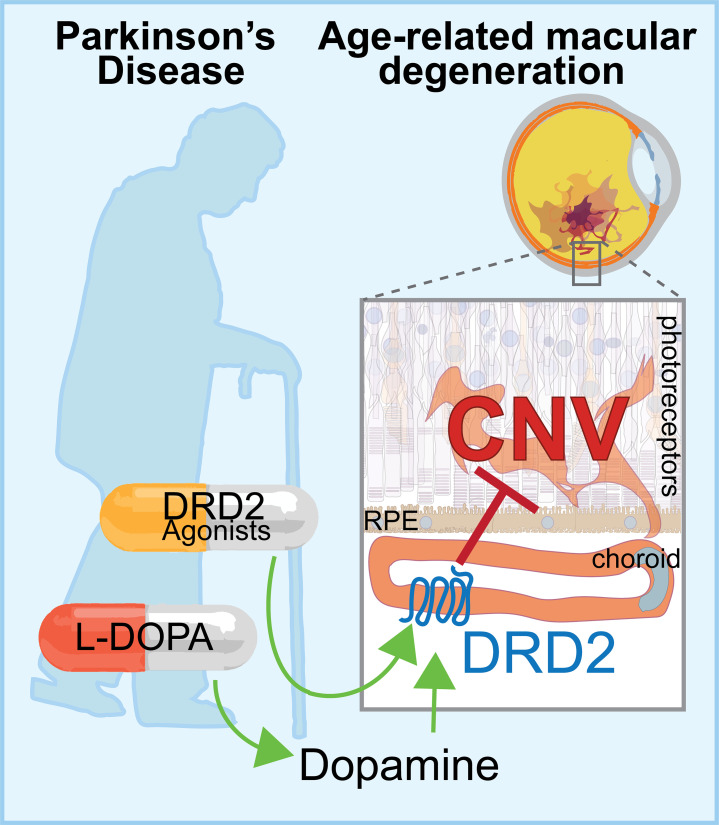

## Introduction

Alongside Alzheimer’s disease, Parkinson’s disease (PD) and age-related macular degeneration (AMD) are the most common age-associated neurodegenerative diseases (1, 2).

AMD is the leading cause of central vision loss in the elderly (3, 4). There are two debilitating late forms of the disease: geographic atrophy, characterized by an extending lesion of both the retinal pigment epithelium (RPE) and the photoreceptor cell layer, and neovascular AMD (nAMD), defined by leaky choroidal neovascularization (CNV) that, if untreated, leads to retinal edema, hemorrhage, and fibrosis (5, 6). The vascular endothelial growth factor (VEGF) is a critical mediator of the sight-threatening neovascular complication that defines nAMD (7, 8), and its inhibition by frequent intravitreal anti-VEGF therapies reduces the risk of legal blindness in patients by 50% (9). However, the continuous need for repeated intravitreal injections of anti-VEGF therapies puts a considerable burden on patients, ophthalmologists, and health care systems (10, 11).

PD is characterized by a progressive, preferential loss of dopaminergic neurons within the substantia nigra pars compacta, resulting in a profound reduction of striatal dopamine, which in turn triggers functional changes in the basal ganglia circuitry and, hence, cardinal motor symptoms including bradykinesia, stiffness, and resting tremor (12, 13). Most newly diagnosed PD patients are treated with a combination of l-DOPA, a metabolic precursor of dopamine, and a DOPA-decarboxylase (DDC) inhibitor (DDI) (14). Differing from dopamine, around 5%–10% of l-DOPA has the ability to traverse the blood-brain barrier. Once inside, an enzyme known as DDC converts it into dopamine, effectively restoring the neurotransmitter levels in the affected brain to alleviate symptoms. To reduce l-DOPA’s peripheral conversion into dopamine, and achieve high l-DOPA/dopamine concentrations in the CNS, it is invariably administered in combination with a peripheral DDI that does not cross the blood-brain barrier (15). Yet, despite the administration of DDI, a significant amount of peripheral l-DOPA is converted into dopamine, increasing plasmatic dopamine levels by 50%–250%, which is responsible for the most common peripheral side effects of this therapy (nausea, vomiting, orthostatic hypotension) (16–18).

Several epidemiological studies have recently shown that the incidence of PD is significantly increased in individuals treated for nAMD (19–21). This link could be due to the repeated anti-VEGF therapy, or to common risk factors (such as the apolipoprotein ε2 variant; ref. 22) or common neurodegenerative mechanisms (23).

On the other hand, one study of retrospective cohorts in large prescription and diagnostic patient databases revealed that patients who were prescribed l-DOPA were protected against AMD and in particular nAMD (24). The authors had previously shown that l-DOPA inhibits the release of VEGF from RPE monocultures in vitro, via the orphan receptor GPR143 (25, 26), and proposed that a similar mechanism inhibits nAMD in patients. However, since l-DOPA treatment is invariably administered with a DDI, it also concomitantly leads to an increase in peripheral dopamine levels (18). Moreover, the vast majority of l-DOPA–treated patients also have PD. Therefore, any of these factors might be responsible for the epidemiological observation of delay and decrease in nAMD incidence.

Using l-DOPA, DDI, and dopamine agonist and antagonist (a) in murine in vivo models of 1-methyl-4-phenyl-1,2,3,6-tetrahydropyridine–induced (MPTP-induced) PD and laser-induced nAMD, (b) in ex vivo choroidal explants, and (c) in in vitro human choroidal endothelial cell cultures, we here investigated the underlying mechanisms of l-DOPA–associated nAMD protection. Our data demonstrated that while MPTP-induced loss of dopaminergic neurons does not directly affect laser-induced CNV, l-DOPA treatment–induced, dopamine-dependent dopamine receptor D2 (DRD2) signaling strongly reduces neovascularization. Mechanistically we demonstrate that dopamine increased the expression of dual-specificity phosphatase 4 (DUSP4), a phosphatase known to deactivate mitogen-activated protein (MAP) kinases involved in angiogenesis. In a retrospective cohort of more than 200,000 patients with nAMD receiving anti-VEGF treatment from the French nationwide insurance database, we further demonstrate that DRD2 agonist–treated PD patients had a significantly delayed age of onset of nAMD and reduced need for anti-VEGF therapies, similar to the effects of the previously reported l-DOPA treatment. Our findings strongly suggest that systemic DRD2 agonists might constitute a much-sought-after therapy to reduce the need for anti-VEGF therapy in patients with nAMD.

## Results

### l-DOPA/DDI treatment prevents choroidal neovascularization independently of subretinal inflammation and the loss of dopaminergic neurons in mice.

A study of retrospective cohorts revealed that nAMD diagnosis is delayed and the frequency of anti-VEGF injections decreased in l-DOPA–treated PD patients (24). However, this could be due to l-DOPA, the coadministered DDI, or the underlying PD pathomechanisms.

**Figure 1:**
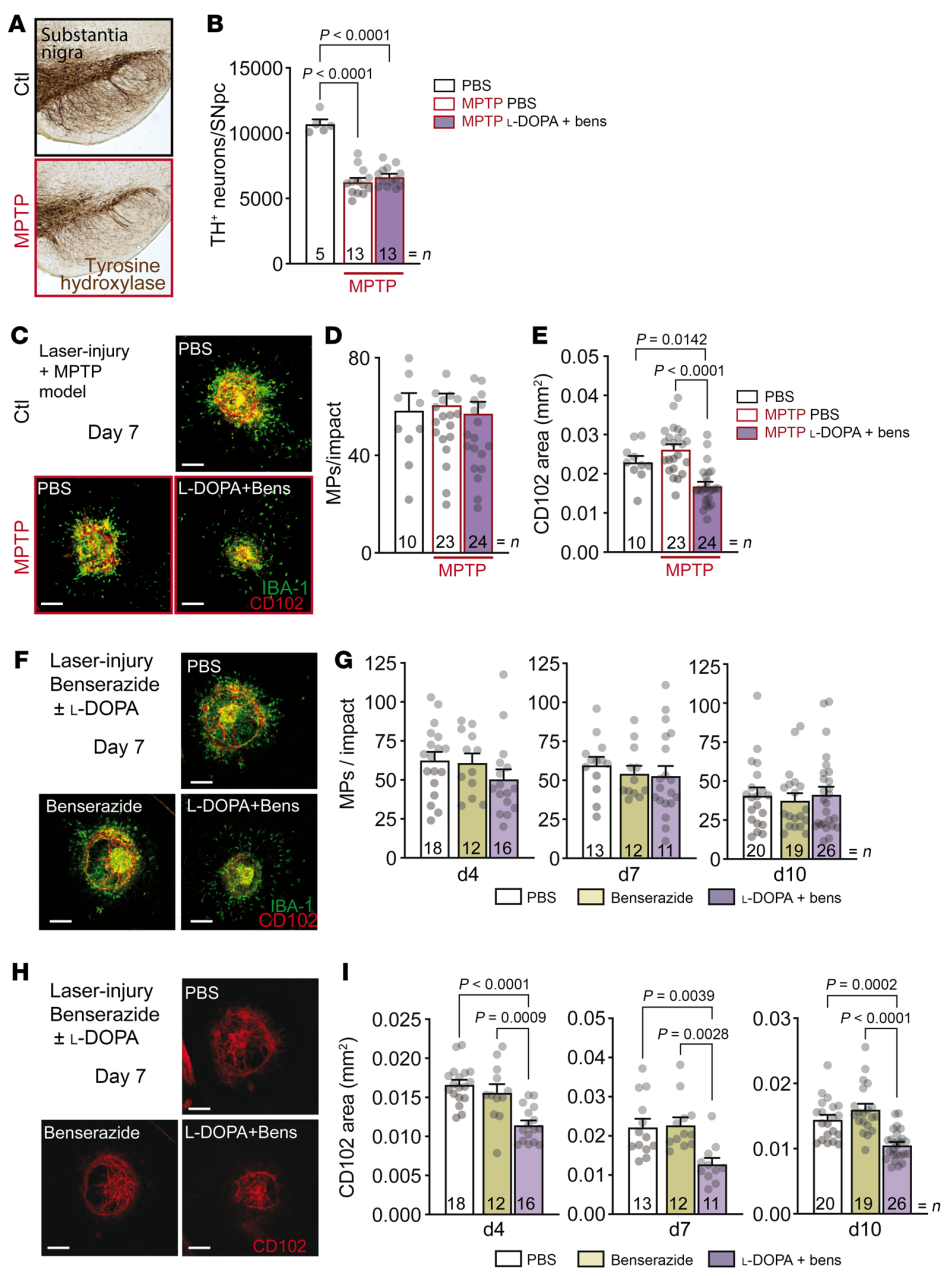
l-DOPA/DDI treatment prevents choroidal neovascularization independently of subretinal inflammation and loss of central dopaminergic neurons in mice. (**A** and **B**) TH-stained SNpc (**A**) and quantification of dopaminergic TH^+^ neurons in SNpc (**B**) of 9-week-old C57BL/6J mice that received PBS or MPTP intoxication at 6 weeks of age and laser injury at 8 weeks and were treated with intraperitoneal PBS or l-DOPA (30 mg/kg/d) plus benserazide (bens) (12 mg/kg/d) from 1 week before injury until 7 days after injury. One-way ANOVA/Bonferroni’s test. (**C**–**E**) IBA-1^–^ (green) and CD102^–^ (red) stained choroidal flat mounts (**C**), quantification of number of subretinal IBA-1^+^ mononuclear phagocytes (MPs) counted on the RPE at a distance of 0–500 μm to CD102^+^ CNV (**D**), and quantification of CD102^+^ CNV area (**E**), 7 days after laser injury of mice that received MPTP intoxication and treatment regimens as for **A**. One-way ANOVA/Bonferroni’s test; control-PBS (Ctl-PBS) vs. MPTP-PBS *P* = nonsignificant. (**F**) IBA-1^–^ (green) and CD102^–^ (red) stained choroidal flat mounts 7 days after laser injury of 8-week-old C57BL/6J mice treated with PBS, benserazide (12 mg/kg/d), or l-DOPA (30 mg/kg/d) plus benserazide (12 mg/kg/d) from 1 week before injury until sacrifice. (**G**) Quantification of subretinal IBA-1^+^ MPs counted on the RPE at a distance of 0–500 μm to CD102^+^ CNV, 4, 7, and 10 days after laser injury of 8-week-old C57BL/6J mice treated with PBS, benserazide (12 mg/kg/d), or l-DOPA (30 mg/kg/d) plus benserazide (12 mg/kg/d) from 1 week before laser injury until sacrifice. One-way ANOVA/Bonferroni’s test; *P* = nonsignificant for each group. (**H**) CD102^–^ (red) stained choroidal flat mounts 7 days after laser injury of 8-week-old C57BL/6J mice treated with PBS, benserazide (12 mg/kg/d), or l-DOPA (30 mg/kg/d) plus benserazide (12 mg/kg/d) from 1 week before injury until sacrifice. (**I**) CD102^+^ CNV area 4, 7, and 10 days after laser injury of 8-week-old C57BL/6J mice treated with PBS, benserazide (12 mg/kg/d), or l-DOPA (30 mg/kg/d) plus benserazide (12 mg/kg/d) from 1 week before injury until sacrifice. One-way ANOVA/Bonferroni’s test. *n* is indicated in each column for each group. Scale bars: 200 μm.

**Figure 2:**
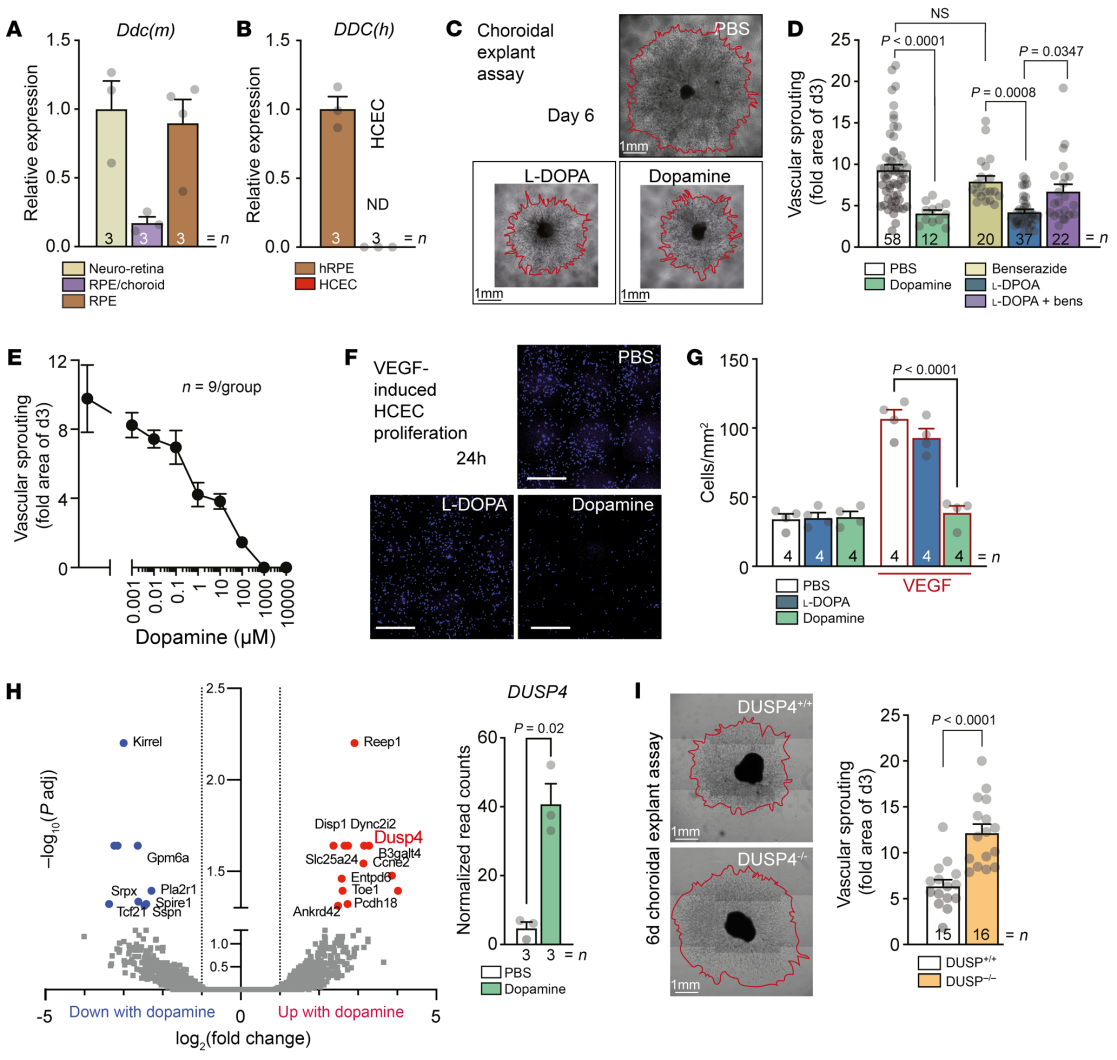
The decarboxylation of l-DOPA to dopamine is necessary for its anti-angiogenic effect on CNV ex vivo and in vitro. (**A**) Quantitative RT-PCR of mouse DOPA-decarboxylase (*Ddc*) mRNAs normalized with β-actin of fresh neuroretina, RPE/choroid, and choroid from 8-week-old C57BL/6J mice. (**B**) Quantitative RT-PCR of human *Ddc* mRNAs normalized with β-actin of fresh human RPE from donor eye and HCECs. (**C**) Representative microphotographs of choroidal explants from 2-week-old C57BL/6J pups at day 6 after 3-day treatment with PBS, l-DOPA (1 μM), or dopamine (1 μM). Scale bars: 1 mm. (**D**) Quantification of vascular sprouting area between days 3 and 6 from choroidal explants prepared from 2-week-old C57BL/6J pups and treated with dopamine (1 μM), benserazide (10 μM), or l-DOPA (1 μM) or preincubated 1 hour with benserazide (10 μM) before addition of l-DOPA (1 μM). One-way ANOVA/Bonferroni’s test. (**E**) Quantification of vascular sprouting area between days 3 and 6 from choroidal explants prepared from 2-week-old C57BL/6J pups and treated with increasing concentrations of dopamine. (**F**) Representative microphotographs of DAPI^+^ HCECs after a 24-hour treatment with PBS, l-DOPA (1 μM), or dopamine (1 μM) with or without VEGF exposure (10 ng/mL). Scale bars: 200 μm. (**G**) Quantification of DAPI^+^ HCECs after a 24-hour treatment with PBS, l-DOPA (1 μM), or dopamine (1 μM) with VEGF exposure (10 ng/mL). One-way ANOVA/Bonferroni’s test, PBS vs. l-DOPA *P* = nonsignificant. (**H**) Volcano plot of differentially expressed genes and scatterplot of DUSP4 read counts of FACS-sorted CD31^+^CD11b^+^ endothelial cells from day 4 choroidal explants that had or had not been incubated with 1 μM dopamine for 24 hours. The volcano plot shows the log_2_(fold change) (*x* axis) versus the significance [–log_10_(*P* value); *y* axis] of the 2,276 genes with a log_2_(fold change) greater than ±1.0. Vertical lines indicate the cutoff of fold change = ±1.0. Adjusted *P* value, indicated for the difference in DUSP4 transcription, was calculated by Benjamini and Hochberg false discovery rate correction method. (**I**) Representative microphotographs and quantification of vascular sprouting area between days 3 and 6 of choroidal explants from 2-week-old C57BL/6J Dusp4^+/+^ and Dusp4^–/–^ pups. Mann-Whitney *U* test. Scale bars: 1 mm. hRPE, fresh human retinal pigment epithelium; ND, not detected. *n* is indicated in each column for each group.

To test whether the loss of dopaminergic neurons or its therapy influences CNV, we subjected C57BL6/J mice to 4 intraperitoneal injections of either a control saline solution or MPTP hydrochloride (20 mg/kg) at 2-hour intervals. This MPTP intoxication regimen reproducibly induced the loss of around 50% of the dopaminergic neurons of the substantia nigra pars compacta (SNpc) as evidenced on tyrosine hydroxylase–immunostained (TH-immunostained) brain sections and by the stereological quantification of TH^+^ neurons of the whole SNpc (Figure 1, A and B), mimicking the pathognomic change underlying PD (27–30). After 1 week, half of the MPTP mice were treated with daily intraperitoneal PBS, while the other half were injected with l-DOPA and benserazide, a commonly used DDI (30 mg/kg/d and 12 mg/kg/d, respectively), in exact analogy to therapeutic regimens for PD. After a week of treatment, the mice underwent subretinal laser injury, which induces subretinal inflammation and choroidal neovascularization and is commonly used as a model of nAMD (6, 31). RPE/choroidal flat mounts stained for IBA-1^+^ mononuclear phagocytes (MPs; green) and CD102^+^ vascular endothelial cells (red; Figure 1C), prepared after an additional 7 days of continued treatment, demonstrated that the number of subretinal infiltrating MPs, a driving force of CNV development (6), was not affected by either the MPTP-induced nigrostriatal lesion or the l-DOPA/benserazide treatment (Figure 1D). However, quantification of the surface covered by CD102^+^ CNV revealed that l-DOPA/benserazide treatment but not the MPTP intoxication induced a significant inhibition of CNV development (Figure 1E). This result suggested that l-DOPA/benserazide treatment and not the dopaminergic pathology induces a reduction in CNV development. Indeed, when we evaluated subretinal IBA-1^+^ MP infiltration (Figure 1, F and G) and CD102^+^ CNV development (Figure 1, H and I) in MPTP-free animals, CNV was significantly inhibited in mice that had been pretreated for 7 days and until sacrifice with l-DOPA/benserazide. This was the case at day 4 after laser injury (maximal MP infiltration), day 7 (maximal CNV development), and day 10 (during CNV involution; ref. 31) (Figure 1H and Figure 2I). In fact, there were no statistical differences between 4, 7, and 10 days in CNV area for mice treated with l-DOPA/benserazide, while there was a significant increase between 4 and 7 days and between 7 and 10 days for mice treated with PBS (data not shown). The MP infiltration was not altered by the treatment at any investigated time points. Importantly, the inhibitory effect was only observed in mice treated with both l-DOPA and benserazide, not in mice treated with the DDI alone.

Taken together, these experiments show that the combined treatment of l-DOPA and benserazide, but not the MPTP-induced degeneration of dopaminergic neurons, nor the inhibition of DDC, inhibits subretinal CNV development. Interestingly, the number of infiltrating MPs was not altered by either the PD model or the treatment at any time point analyzed, in contrast to the proinflammatory effect of MPTP intoxication and l-DOPA treatment in the brain (32, 33).

### The decarboxylation of l-DOPA to dopamine is necessary for the anti-angiogenic effect on CNV ex vivo and in vitro.

Our in vivo results demonstrate that PD-associated l-DOPA and benserazide treatment, but not benserazide alone, exerts an inhibitory effect on CNV formation, without affecting MP infiltration. The inhibition of CNV in vivo could therefore be mediated by l-DOPA, or through dopamine, which is increased (by 50%–250% in patients [ref. 18] and up to 200% in animal models [ref. 34]) with systemic l-DOPA/benserazide treatment of PD patients. This is due to the fact that benserazide only partially prevents the conversion of the high therapeutic doses of l-DOPA to dopamine by the DDC in vivo, likely owing to only partial DDC inhibition in peripheral dopaminergic neurons.

In the eye, *Ddc* mRNA expression was detected in the retina, but also in freshly prepared mouse RPE as well as in the RPE/choroid, where it is additionally expressed in sympathetic neurons and mast cells to produce adrenaline and histamine, respectively (Figure 2A). It was also expressed in fresh human RPE (Figure 2B), in accordance with previous reports of mRNA and protein expression of DDC in the RPE (35). In contrast, *Ddc* mRNA was undetectable in human choroidal endothelial cells (HCECs) compared with RPE (Figure 2B). Interestingly, dopamine and l-DOPA significantly inhibited vascular sprouting from choroidal explants compared with controls, when administered from day 3 to day 6 (Figure 2C). Quantifications of the sprouting area, expressed as the increase of sprouting area between 3 and 6 days, revealed a significant inhibition by dopamine and l-DOPA at 1 μM, while benserazide alone had no significant effect (Figure 2D). Importantly, prior administration of benserazide (10 μM) led to a much attenuated anti-angiogenic effect of l-DOPA in the choroidal explants (Figure 2D). These results suggested that preincubation with 10 μM benserazide achieved a near-complete DDC inhibition and diminished dopamine production in the explants. Indeed, dopamine in turn dose-dependently inhibited vascular sprouting (Figure 2E). The results from the choroidal explants suggested that l-DOPA had to be converted to dopamine for its anti-angiogenic effect. Similarly, dopamine significantly inhibited VEGF-induced proliferation of HCECs in vitro (Figure 2, F and G). l-DOPA, which cannot be metabolized to dopamine in HCECs that lack DDC (Figure 2B), did not.

A comparison by bulk RNA-Seq of FACS-sorted CD31^+^CD11b^–^ endothelial cells from day 4 choroidal explants that had or had not been incubated with 1 μM dopamine from day 3 to day 4 revealed 13 significantly upregulated (red dots) and 9 significantly downregulated transcripts (blue dots) (Figure 2H). Among the significantly upregulated transcripts was dual-specificity phosphatase 4 (DUSP4), a phosphatase known to deactivate the mitogen-activated protein (MAP) kinases ERK1, ERK2, and JNK (36, 37), all well known for their implication in pro-proliferative VEGF signaling and angiogenesis (38). Indeed, vascular sprouting from Dusp4^–/–^ choroidal explants exhibited a significant increase in comparison with Dusp4^+/+^ choroidal explants, thus affirming its role in choroidal endothelial cell proliferation (Figure 2I).

Taken together, our data showed that dopamine prevents VEGF-induced HCEC proliferation in vitro and dose-dependently inhibits CNV ex vivo. l-DOPA needs to be converted into dopamine to have a similar effect, as it had no antiproliferative impact on DDC-free HCECs, and its effect in choroidal explants was inhibited when the DDC was pharmacologically inactivated. Mechanistically, dopamine increased the expression of the phosphatase DUSP4 in choroidal endothelial cells ex vivo, which has been shown to inhibit pro-angiogenic signaling, as confirmed by the observation that the vascular sprouting of Dusp4^–/–^ choroidal explants was significantly increased in comparison with controls.

### DRD2 activation mediates the CNV inhibition observed under l-DOPA/DDI treatment in vivo.

Dopamine mediates its biological effects through 2 subfamilies of G protein–coupled dopamine receptors: DRD1 and DRD5 (D1-like), and DRD2, DRD3, and DRD4 (D2-like) (39). All dopamine receptors, except DRD3, are expressed in the retina and the RPE. These receptors mediate a broad range of functions, including the dopamine-regulated processes from neurotransmission (DRD5 and DRD4), circadian cycle (DRD2/DRD4), phagocytosis (DRD1), and the regulation of eye growth (balance of DRD5 and DRD4 activation), among others (40–44). Beyond the eye, dopamine receptors are expressed on tumor immune cells and endothelial cells (45–47), but their expression and function in CNV are unknown.

**Figure 3:**
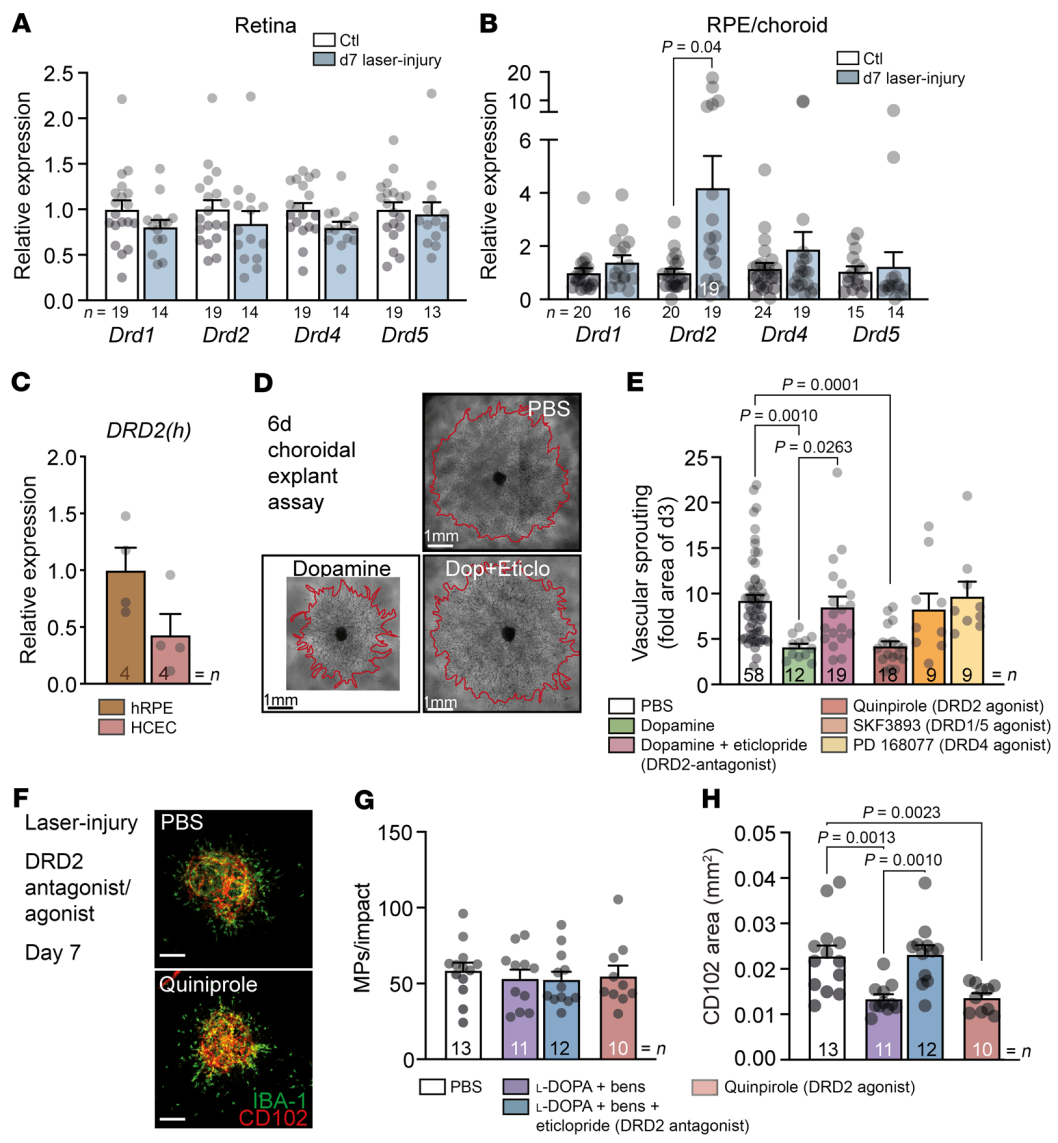
DRD2 activation mediates the CNV inhibition observed under l-DOPA/DDI treatment in vivo. Quantitative RT-PCR of dopamine receptor (DR1–DR5) mRNAs normalized with β-actin of 8-week-old C57BL/6J mouse retina (**A**) or choroid (**B**) 7 days after laser injury compared with control mice. Mann-Whitney *U* test DRD2 in CNV group vs. DRD2 in Ctl group; *P* value indicated in the figure when statistically significant. (**C**) Quantitative RT-PCR of DRD2 mRNAs normalized with β-actin of fresh RPE from donor eye and HCECs. (**D**) Representative microphotographs of choroidal explants of 2-week-old C57BL/6J pups at day 6 after 3-day treatment with PBS or dopamine (1 μM) or pretreated 1 hour with eticlopride (10 μM) before addition of dopamine (1 μM). Scale bars: 1 mm. (**E**) Quantification of choroidal vascular sprouting from 2-week-old C57BL/6J pups at day 6 after 3 days of treatment with PBS or dopamine (1 μM) or pretreated for 1 hour with eticlopride (10 μM) before addition of dopamine (1 μM), quinpirole (10 μM), SKF38393 (10 μM), or PD168077 (10 μM). One-way ANOVA/Bonferroni’s test; *P* values indicated in the figure, PBS vs. SKF38393 and PD168077 *P* = nonsignificant. (**F**) IBA-1^–^ (green) and CD102^–^ (red) stained choroidal flat mounts 7 days after laser injury of 8-week-old C57BL/6J mice treated with PBS or quinpirole (5 mg/kg) from 1 week before injury until sacrifice. Scale bars: 200 μm. (**G**) Quantification of subretinal IBA-1^+^ MPs counted on the RPE at a distance of 0–500 μm to CD102^+^ CNV 7 days after laser injury of 8-week-old C57BL/6J mice and treated with PBS, l-DOPA (30 mg/kg) plus benserazide (12 mg/kg), l-DOPA (30 mg/kg) plus benserazide (12 mg/kg) plus eticlopride (1 mg/kg), or quinpirole (5 mg/kg) from 1 week before injury until sacrifice. One-way ANOVA/Bonferroni’s test; *P* = nonsignificant for each group. (**H**) CD102^+^ CNV area 7 days after laser injury of 8-week-old C57BL/6J mice and treated with PBS, l-DOPA (30 mg/kg) plus benserazide (12 mg/kg), l-DOPA (30 mg/kg) plus benserazide (12 mg/kg) plus eticlopride (1 mg/kg), or quinpirole (5 mg/kg) from 1 week before injury until sacrifice. One-way ANOVA/Bonferroni’s test; *P* values indicated in the figure. hRPE, fresh human retinal pigment epithelium. *n* is indicated in each column for each group.

RT-PCR analysis of mRNA extracts from retina and choroid of control mice and mice 7 days after laser injury confirmed that all dopamine receptors except DRD3 (not detected) were present in retina and choroid (Figure 3, A and B) as previously reported (40, 48, 49). DRD2 mRNA in the RPE/choroid was the only transcript induced by laser injury, while its expression in the retina and all other dopamine receptors remained stable (Figure 3, A and B). DRD2 was also detectable in both fresh human RPE cells (where it can stem from RPE expression but also ingested rod outer segments) and HCECs, the 2 major cell types of RPE/choroidal preparations (Figure 3C). Functionally, the specific DRD2 antagonist eticlopride completely reversed the anti-angiogenic effect of dopamine on the vascular outgrowth of RPE/choroidal explants (Figure 3, D and E). Moreover, the activation of DRD2 with the specific DRD2 agonist quinpirole was sufficient to achieve a comparable anti-angiogenic effect to that of dopamine. Yet activation of DRD1 and DRD5 (SKF38393) or DRD4 (PD168077) had no such effect (Figure 3E). In vivo, the DRD2 antagonist eticlopride also entirely reversed the l-DOPA/DDI–induced inhibition of CD102^+^ CNV, quantified on IBA-1/CD102–double-labeled RPE/choroidal flat mounts of mice 7 days after laser injury (Figure 3, F–H). Most importantly, the DRD2 agonist quinpirole, administered in exact analogy to therapeutic regimens of PD (5 mg/kg/d) (50), achieved a comparable anti-angiogenic effect to the l-DOPA/DDI treatment (Figure 3G), and none of the administered dopamine receptor agonists and antagonists significantly altered the number of infiltrating IBA-1^+^ MPs (Figure 3H).

Taken together, our data show that blocking the DRD2 receptor prevents the anti-angiogenic effect of dopamine ex vivo and l-DOPA/DDI treatment in vivo, demonstrating that l-DOPA/DDI treatment’s anti-angiogenic effect requires the activation of DRD2. Importantly, we show that DRD2 activation is sufficient to produce a similar therapeutic response.

### The incidence of nAMD and frequency of anti-VEGF injections are reduced in PD patients treated with l-DOPA or DRD2 agonists.

A retrospective cohort study from large prescription and diagnostic patient databases revealed that nAMD is delayed in PD patients treated with l-DOPA (24). Because of the relatively low prevalence of PD (1% of subjects older than 60 years) and AMD (3% of subjects older than 70 years for late-stage AMD, nAMD, or geographic atrophy), even large observational studies are not sufficiently powered to establish the association between AMD, PD, and its treatment by l-DOPA and DDIs (1, 2).

**Figure 4:**
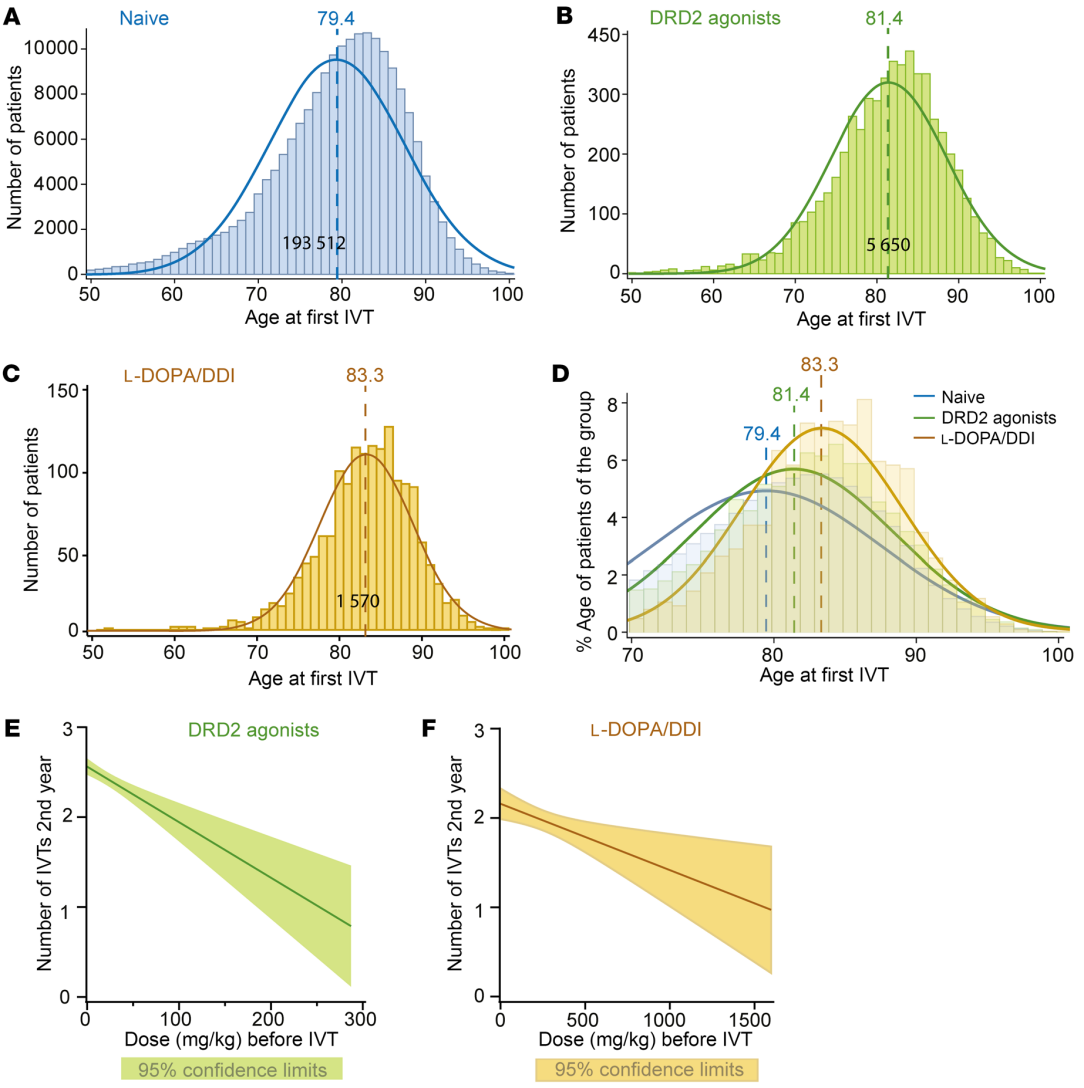
Incidence of nAMD and frequency of anti-VEGF injections are reduced in PD patients treated with l-DOPA/DDI and DRD2 agonists. (**A**–**C**) Number of patients (*y* axis) receiving the first IVT as a function of age (*x* axis) of the 193,512 nAMD control patients (**A**), of the 5,650 parkinsonian nAMD patients receiving DRD2 agonists (**B**), and of the 1,570 parkinsonian nAMD patients receiving l-DOPA/DDI therapy (**C**). The mean age for each group is indicated by the dotted line. (**D**) Distribution curves normalized for each group (percentage of cases of the group) of patients receiving the first IVT as a function of age (ANOVA test; control patients with nAMD vs. DRD2 agonist or l-DOPA/DDI nAMD patients *P* < 0.0001). (**E** and **F**) Correlation of DRD2 agonist (**E**) or l-DOPA/DDI (**F**) treatment group with the number of IVTs during the second year of nAMD treatment (anti-VEGF–naive patients with nAMD in France are generally initiated with a fixed proactive regimen during the first year and the treatment regimen is only then adapted to pathology activity) (univariate linear generalized model; control patients with nAMD vs. DRD2 agonist nAMD patients only, *P* < 0.0001 [**E**], or vs. l-DOPA/DDI nAMD patients only, *P* = 0.02 [**F**]).

To overcome this problem, we data-mined the French national health information database (Système National des Données de Santé [SNDS]), which contains information regarding all treatments, procedures, and diagnostics for the 65 million people treated in France. We first extracted data on patients with nAMD registered between January 2008 and December 2016 with a minimum 2 years of follow-up after their first intravitreal therapy (IVT) with anti-VEGF, on a database running from 2007 to 2018. Patients with nAMD were identified as being aged over 50, having nAMD disease code on hospitalization discharge or nAMD long-term disease, and having had nAMD treatment (anti-VEGF IVT, or dynamic phototherapy with verteporfin) in the absence of treatment for other retinal neovascular disease (dexamethasone implant, retinal photocoagulation, etc.). Other exclusion criteria were high myopia, diagnosis of other retinal diseases, treatments for other macular diseases (dexamethasone implant, retinal photocoagulation, etc.), or other retinal conditions. The data extracted were age at the first anti-VEGF IVT; sex; diabetic, hypertensive, dyslipidemia, chronic alcoholism, smoking, and obesity status; number of IVTs performed in the first year, in the second year, and over the first 2 years; and therapeutic class(es) of anti-parkinsonian treatment and anti-emetics. The daily dose of anti-parkinsonian treatment and anti-emetics was calculated from the frequency and dosage of drugs prescribed. Naive patients with nAMD were defined as those naive of any anti-parkinsonian treatment before their first IVT. During the 9-year period, 202,629 patients were treated for nAMD by anti-VEGF injections (see patient characteristics in Table 1). A total of 9,117 patients were treated with drugs acting directly on the dopaminergic system; of these, 1,570 patients were on l-DOPA/DDI, and 5,650 were treated with DRD2 agonists only (the remaining 1,897 patients were treated with either a combination of l-DOPA/DDI and DRD2 agonists, or other dopaminergic drugs). They were compared with the 193,512 patients with nAMD naive of any dopaminergic treatments, serving as control. Figure 4, A–C, depicts the number of patients receiving the first IVT as a function of their age in each group.

Our data confirm that patients treated with l-DOPA/DDI were significantly older than control patients when receiving their first anti-VEGF injection for nAMD (Figure 4D; i.e., date of diagnosis of nAMD) (83.3 [± 5.6] vs. 79.4 [± 8.1] years old, respectively, ANOVA *P* < 0.0001). Interestingly, despite lower dosages of DRD2 agonists compared with l-DOPA, patients treated with DRD2 agonists only were also significantly older at first anti-VEGF injection than control patients (Figure 4D; 81.4 [± 7.0] vs. 79.4 [± 8.1] years old, respectively, ANOVA *P* < 0.0001). Moreover, increasing daily doses of DRD2 agonist were associated with an increase in age at IVT onset, +0.007 yr/mg/d (univariate generalized linear model, *P* = 0.01).

To address the question of the potential influence of increasing use of l-DOPA/DDI or DRD2 agonists as the population ages during the study period, we divided our cohort into 3 time periods according to the beginning of anti-VEGF treatment: 2008–2010, 2011–2013, and 2014–2016. As was previously shown (51), we first confirmed an increase in age at first IVT between the first period (2008–2010) and the second period (2011–2013: +0.5 years, 95% CI 0.41–0.58) and between the first period and the third period (2014–2016: +1.88 years, 95% CI 1.79–1.98) (multivariate generalized linear model, *P* < 0.0001). It should be noted, however, that anti-parkinsonian drug delivery remained stable over years, as reported on the Open Medic database, containing information on every drug delivery in France since 2014 (https://www.data.gouv.fr/en/datasets/open-medic-base-complete-sur-les-depenses-de-medicaments-interregimes/).

In multivariate analysis (accounting for sex, high blood pressure, diabetes, dyslipidemia, chronic alcoholism, smoking, obesity, IVT initiation period, and other treatment acting indirectly on the dopaminergic system), the positive association with older age at first IVT remained significant in patients treated with l-DOPA/DDI (multivariate generalized linear model, *P* < 0.0001) or DRD2 agonists (multivariate generalized linear model, *P* < 0.0001).

Together, these findings suggest that later age of first IVT injections in PD patients is independently associated with l-DOPA/DDI treatment or DRD2 agonists. Specifically, it was not due to increase in age at first IVT injections in AMD patients, nor to anti-parkinsonian drug delivery over years.

Next, we investigated whether l-DOPA/DDI and DRD2 agonists influenced anti-VEGF IVT frequency dose-dependently. We focused our analysis on the second year, as anti-VEGF–naive patients with nAMD are generally initiated with a fixed proactive regimen during the first year, including a loading dose every 3 months. Therefore, IVT frequency might only truly reflect pathology activity in the second year of treatment. Our data show that the number of intravitreal anti-VEGF injections needed in the second year decreased by 0.6 injections per 100 mg/d daily dose of DRD2 agonists (Figure 4E; univariate generalized linear model, *P* < 0.0001). Similarly, the number of intravitreal anti-VEGF injections decreased by 0.13 injections per 100 mg/d of l-DOPA treatment in the second year (Figure 4F; univariate generalized linear model, *P* = 0.02).

Taken together, our data confirm that l-DOPA/DDI therapy delays nAMD onset. Furthermore, we demonstrate that DRD2 agonists delay disease onset similarly. Most importantly, our data show that both treatments dose-dependently reduce the frequency of anti-VEGF IVTs needed to maintain nAMD stability.

## Discussion

An analysis of retrospective cohorts in large prescription and diagnostic patient databases previously revealed that patients prescribed l-DOPA/DDI, the majority diagnosed with PD, were somewhat protected against AMD, and in particular nAMD (24). However, these interesting findings did not determine whether l-DOPA, concomitantly prescribed DDIs, or PD itself was responsible for the observed association.

In mice, CNV formation can be induced by laser injury (31), and MPTP intoxication induces the loss of dopaminergic neurons in the substantia nigra pars compacta, mimicking the pathognomic change underlying PD (27–30). Our experiments show that MPTP-induced nigrostriatal pathway injury does not alter a subsequent laser-induced subretinal inflammatory reaction or CNV formation. However, when treated with a combination of l-DOPA and DDI, in an exact analogy with PD patients, laser-injured parkinsonian mice exhibited reduced CNV but a similar MP infiltration rate. These results strongly suggest that the PD-associated l-DOPA therapy, and not the loss of central dopaminergic neurons, accounted for the CNV inhibition. Indeed, when mice without MPTP intoxication were treated with the combination therapy of l-DOPA/DDI, we found the subretinal angiogenesis, but not the MP infiltration, to be similarly inhibited. The DDI alone had no such effect.

l-DOPA is converted to dopamine by the aromatic l-amino acid decarboxylase, which is commonly called the DDC despite the fact that it has a variety of other substrates and is involved in the synthesis of other neurotransmitters and neuromodulators as well as the production of histamine (52). Unlike dopamine, 5%–10% of l-DOPA can cross the blood-brain barrier to reach the brain parenchyma, where a DDC converts it into dopamine. In PD patients, l-DOPA treatment is invariably accompanied by a peripheral DDI (15) to limit peripheral l-DOPA conversion into CNS-impenetrable dopamine, thereby keeping peripheral l-DOPA concentrations at a high rate and favoring l-DOPA penetration to the CNS. However, despite the administration of DDI, a significant amount of therapeutically administered l-DOPA is converted into dopamine, increasing the plasma dopamine levels by 50%–250%, which is responsible for most common peripheral side effects (nausea, vomiting, orthostatic hypotension) (16–18, 34).

In our model, the inhibitory effect of the l-DOPA/DDI treatment could therefore be directly due to l-DOPA itself or to dopamine. Increased dopamine might arise from retinal (observed in treated PD patients) (53) or choroidal conversion of l-DOPA to dopamine, additionally to the above-described increase in plasma levels. In RPE/choroidal explants, l-DOPA inhibited vascular sprouting only in the presence of DDC activity, which, as we show, is expressed in the RPE/choroid tissue (35). These results demonstrate that l-DOPA needs to be converted into dopamine to display such inhibitory effect ex vivo. Accordingly, l-DOPA did not inhibit the VEGF-induced proliferation of HCECs, a cell line that does not express DDC and is therefore unable to convert l-DOPA into dopamine. Dopamine, on the other hand, dose-dependently inhibited vascular sprouting from choroidal explants and VEGF induced HCEC proliferation. Mechanistically, we showed that dopamine increased the expression of the phosphatase DUSP4 in choroidal endothelial cells and that vascular sprouting of Dusp4^–/–^ choroidal explants was significantly increased in comparison with controls. DUSP4 is well known to deactivate the MAP kinases ERK1, ERK2, and JNK (36, 37), all well known for their implication in pro-proliferative VEGF signaling and angiogenesis (38). The dopamine-induced overexpression of DUSP4 might thereby interfere with VEGF signaling and mediate the inhibition of the vascular sprouting.

Taken together, these ex vivo and in vitro data showed that dopamine, and not its precursor l-DOPA, inhibits HCEC proliferation and choroidal angiogenesis. Dopamine exerts its biological effects via dopamine receptors that cannot be activated by l-DOPA (54). Interestingly, DRD2, which is expressed in HCECs, is the only dopamine receptor whose expression is increased in laser-induced proliferation of choroidal endothelial cells. Pharmacological blockade of this receptor using the highly specific antagonist eticlopride (55, 56) completely inhibited the dopamine-dependent anti-angiogenic effect on choroidal vascular sprouting. These results and the observation that a highly selective DRD2 agonist, quinpirole (57, 58), but not other dopamine receptor–specific agonists, was able to mimic the anti-angiogenic potential of dopamine strongly suggest that DRD2 almost exclusively mediates dopamine anti-angiogenic activity. Although this is, to our knowledge, the first report showing that DRD2 activation potently mitigates CNV, quinpirole (a DRD2/3 agonist) and eticlopride (a DRD2 antagonist) have been shown to inhibit tumoral angiogenesis and to promote neovascularization in dermal wound healing (45, 59, 60). Indeed, DRD2 activation by dopamine has been shown to inhibit endothelial cell proliferation, as it blocks VEGFR2 phosphorylation and subsequent downstream signaling (45, 46, 61). These observations suggest that it is likely the activation of DRD2 on vascular endothelial cells that inhibits CNV in our models, although we do not exclude the possibility that DRD2 activation on RPE cells or subretinal MPs represses the release of angiogenic factors.

Most importantly, when we coadministered the DRD2 antagonist eticlopride with l-DOPA/DDI in laser-injured mice in vivo, we completely abolished the anti-angiogenic effect of the PD treatment. This result confirms that the anti-angiogenic effect of l-DOPA/DDI treatment that we observed in vivo in experimental mice requires the conversion of l-DOPA to dopamine and the activation of the DRD2 receptor.

l-DOPA has been shown to directly activate the receptor GPR143 independently of dopamine and to inhibit VEGF secretion in vitro (25, 26). However, in our ex vivo choroidal explant model and in laser-induced choroidal neovascularization, this mechanism did not seem to play a detectable role, as the conversion of l-DOPA to dopamine was necessary for the anti-angiogenic effect.

Taken together, our data showed that dopamine prevents VEGF-induced HCEC proliferation in vitro and dose-dependently inhibits CNV ex vivo. l-DOPA needs to be converted into dopamine to hold a similar activity, as it had no antiproliferative impact on DDC-free HCECs, and its effect in choroidal explants was inhibited when the DDC was pharmacologically inactivated.

Finally, we show that the administration of the DRD2 agonist quinpirole as a monotherapy was sufficient to significantly inhibit pathogenic subretinal CNV.

In the human disease, our analysis of the national insurance database of more than 200,000 patients treated by anti-VEGF IVT confirms that patients with PD and treated with l-DOPA/DDI were significantly older at the beginning of treatment for nAMD and required fewer anti-VEGF injections than other patients. Importantly, this was also true for DRD2 agonists, a finding that could not specifically be addressed in a previous study (24). Furthermore, we could study the dose effect of treatment and consider interaction between treatments. Importantly, both treatments, DOPA/DDI and DRD2 agonists, dose-dependently reduced the number of anti-VEGF IVTs. It should be noted that anti-VEGF–naive patients with nAMD in France are generally initiated with a fixed proactive regimen during the first year, and the treatment regimen is only then adapted to pathology activity, explaining why we analyzed only the second year of treatment. The relatively low number of injections realized in the second year is in accordance with recent national cohort studies.

Taken together, our findings show that DRD2 agonists increase the age at first IVT by 0.7 years and reduce the IVT frequency by 0.6 injections per year (second year) per 100 mg/d of drug taken. Such a strong dose-dependent effect supports our observations in mouse models where DRD2-specific agonists are highly effective.

We acknowledge several limitations. The results presented in the pharmacoepidemiological study should be considered in light of the potential confounding factors. Many of these were taken into account in the multivariate analyses and are associated with the occurrence of exudative AMD or PD, or are known to modulate the dopaminergic system. However, other confounding factors, such as the date of PD diagnosis, cannot be obtained in the database. Moreover, there can be an unexpected association with reduced ophthalmological care utilization in patients with advanced PD. We accounted for this by focusing on patients treated with monotherapy with dopaminergic agonists or l-DOPA, which constitute the first-line treatment for PD. These patients received, on average, moderate doses, indicative of early-stage disease. While a marginal delay in treatment initiation due to PD-related care-access challenges might explain a slight shift in the timing of the first injection or injection frequency, it does not explain a delay of several years for a condition necessitating urgent and singular anti-VEGF treatment.

In general, it is important to note that the health insurance databases, such as the SNDS, are administrative databases that contain a limited amount of information for each patient (disease codes, consumption of care, but not disease onset or disease severity). Therefore, case identification was based on treatment delivery and correct coding of hospitalizations and long-term diseases. Compared with retrospective clinical studies based on patients’ hospital files, the available information is therefore limited. On the other hand, pharmacoepidemiological studies, owing to their nationwide nature and great number of patients, are sufficiently powered to detect associations of diseases and treatments that are otherwise difficult to detect. Here, we combined the pharmacoepidemiological study with animal models that closely mimic the diseases to evaluate the plausibility of the associations and possible causalities.

Together, our experimental and epidemiological work strongly suggests that l-DOPA treatment, and not PD disease or concomitant DDI therapy, reduces the need for anti-VEGF IVTs in patients with nAMD. Furthermore, our data demonstrate that l-DOPA exerts its protective effect via DRD2 activation following DDC-dependent conversion into dopamine in animal models, and that DRD2 activation in patients similarly reduces IVT burden in patients with nAMD. Preventive systemic administration of DRD2 agonists has the potential to delay the progression of high-risk intermediate AMD patients to nAMD and to reduce their continuous need for repeated intravitreal anti-VEGF injections.

## Methods

### Sex as a biological variable.

Male mice were used to minimize variations in angiogenesis due to reproductive hormonal changes observed in females.

### Animals.

Wild-type (C57BL/6J) male mice were obtained from Janvier Labs. Dusp4^–/–^ mice were generated as originally outlined by coauthor R. Plevin and colleagues (62). They were kept under specific pathogen–free conditions in a 12-hour light/12-hour dark cycle and had access to food and water ad libitum. Upon arrival, mice were acclimated for at least 1 week before any experimentation.

### Chemicals/treatments.

MPTP hydrochloride, l-3,4-dihydroxyphenylalanine methyl ester, and benserazide hydrochloride were purchased from Sigma-Aldrich. Dopamine, quinpirole, SKF38393, PD168077, and eticlopride were purchased from Tocris Bioscience. Human recombinant VEGF165 was purchased from R&D Systems.

### MPTP model.

For all experiments, the acute MPTP model was used as previously described (30). Briefly, male mice received 4 intraperitoneal injections of either a control saline solution or MPTP hydrochloride (20 mg/kg; Sigma-Aldrich) at 2-hour intervals and were kept for 48 hours at 28°C before being returned to 22°C for the rest of the experiment. MPTP intoxication resulted in mortality rates between 10% and 30%. TH^+^ neurons were quantified stereologically using the VisioScan stereology tool on regularly spaced 20-μm-thick sections covering the whole substantia nigra pars compacta (SNpc). The SNpc was delineated (63), and TH^+^ cell bodies were counted by bright-field microscopy, using a Leitz microscope equipped with Mercator image analysis software (ExploraNova).

### Laser injury model.

Eight-week-old male mice were anesthetized with intraperitoneal injection of ketamine (100 mg/kg) and xylazine (10 mg/kg). After pupil dilation, laser coagulations were performed with a 532 nm ophthalmological laser Yag Eyelite (Vitra Laser, Alcon) mounted on an operating microscope. Four impacts per eye were performed for immunochemistry assays, and 10–12 impacts per eye were performed for quantitative PCR assays (450 mW, 50 milliseconds, and 250 μm). One week before the laser injury and until sacrifice, the mice were treated with daily intraperitoneal injections of PBS (control), l-DOPA (30 mg/kg/d)/benserazide (12 mg/kg/d) (28, 64), quinpirole (5 mg/kg/d) (50), or eticlopride (1 mg/kg/d) (65, 66). The concentrations used in mice closely matched the concentrations used in the clinic.

### Immunohistochemistry and histology.

After fixation in a 4% paraformaldehyde solution, the retinas and choroids were incubated with anti–IBA-1 (1:400; 019-19741, FUJIFILM Wako) and anti-CD102 (for laser experiments only; 1:200; 553325, BD Biosciences Pharmingen) in PBS containing 0.1% Triton X-100 overnight at room temperature with gentle rocking. After a few washes in PBS, samples were incubated for 2 hours at room temperature with appropriate Alexa Fluor–conjugated secondary antibodies (1:500; Thermo Fisher Scientific) in PBS solution and were counterstained with Hoechst (1:1,000; 33258, Thermo Fisher Scientific). Preparations were rinsed and mounted on glass slides with Fluoromount aqueous mounting medium (Sigma-Aldrich). Preparations were observed under a fluorescence microscope (DM5500, Leica), and the surface covered by CD102^+^ CNV was measured on photographs. The average CNV size was calculated; IBA-1^+^ MPs on the RPE were counted in a diameter of 500 μm around the CD102^+^ neovascularizations, and IBA-1^+^ cells were counted on whole RPE/choroidal flat mounts and on the outer segment side of the retina.

Deeply anesthetized mice were perfused with PBS, followed by 100 mL of 4% paraformaldehyde to fix the brain tissue. The brains were dissected and stained with monoclonal anti-mouse tyrosine hydroxylase (1:200; 22941, Immunostar).

### Reverse transcription and RT-PCR.

Total RNA was isolated with Nucleospin RNAII (Macherey-Nagel). RNA yields were then measured at 260 nm using the NanoDrop 8000 spectrophotometer (Thermo Fisher Scientific). Single-stranded cDNA was synthesized using 1 μg of total RNA pretreated with amplification-grade DNase, oligo-dT as primer, and SuperScript II reverse transcriptase (Thermo Fisher Scientific). RT-PCR was realized by the StepOne Plus RT-PCR system (Applied Biosystems), using 1:100 of cDNA incubated with the polymerase and the appropriate amounts of nucleotides (PowerSYBR Green PCR mix, Applied Biosystems). PCR reactions were performed in 45 cycles of 15 seconds at 95°C and 45 seconds at 60°C. Results were normalized by expression of β-actin. Primers were purchased from Integrated DNA Technologies Inc. Sequences are as follows:

DDC(h) forward 5′-TGGGGACCACAACATGCTG-3′; DDC(h) reverse 5′-TCAGGGCAGATGAATGCACTG-3′; DRD2(h) forward 5′-GAATTTCCACTCACCTACCACC-3′; DRD2(h) reverse 5′-CAACGGGTCAGACGGGAAG-3′; β-actin(h) forward 5′-GCACTCTTCCAGCCTTCCTT-3′; β-actin(h) reverse 5′-CTTCTGCATCCTGTCGGCAA-3′; Ddc(m) forward 5′-AGCTGACTATCTGGATGGCAT-3′; Ddc(m) reverse 5′-ACCCCTGGCATGATTATCTTCT-3′; Drd1(m) forward 5′-ATGGCTCCTAACACTTCTACCA-3′; Drd1(m) reverse 5′-GGGTATTCCCTAAGAGAGTGGAC-3′; Drd2(m) forward 5′-CCCTGGGTCGTCTATCTGGAG-3′; Drd2(m) reverse 5′-GCGTGTGTTATACAACATAGGCA-3′; Drd4(m) forward 5′-GCCTGGAGAACCGAGACTATG-3′; Drd4(m) reverse 5′-CGGCTGTGAAGTTTGGTGTG-3′; Drd5(m) forward 5′-CTCGGCAACGTCCTAGTGTG-3′; Drd5(m) reverse 5′-AATGCCACGAAGAGGTCTGAG-3′; β-actin(m) forward 5′-AAGGCCAACCGTGAAAAGAT-3′; β-actin(m) reverse 5′-GTGGTACGACCAGAGGCATAC-3′.

### Vascular sprouting from choroid ex vivo.

Experiments were performed as previously described (67, 68). Eyes were enucleated from 2-week-old C57BL/6J pups and Dusp4^–/–^ mice (62) and kept in ice-cold Opti-MEM Reduced Serum Media (Invitrogen) before dissection. The choroid-scleral complex was separated from the other eye tissues and cut into approximately 1 mm × 1 mm fragments. Choroid/sclera fragments were placed in growth factor–reduced phenol red–free Matrigel (Corning) seeded in 96-well plates.

Choroid/sclera fragments were then cultured for 3 days in Endothelial Cell Growth Medium 2 (ECGM-2; Promocell GmbH) supplemented with 1% penicillin/streptomycin in a 37°C cell culture incubator. On day 3, choroid fragments were treated with specific molecules as described from day 3 until day 6 of culture. Photos of individual explants were taken, and the areas of sprouting out of the explant were quantified with ImageJ software (NIH) at day 3 and day 6. The surface of each individual choroidal explant and preincubation sprouts at day 3 was subtracted from the surface at day 6 and divided by the preincubation sprouts at day 3 to calculate the vascular sprouting that occurred in the presence or absence of specific treatment as a fold of day 3 sprouting.

### RNA-Seq analysis of choroidal endothelial cells from dopamine-stimulated choroidal explants.

Choroid/sclera fragments were cultured as described above. On day 3, choroid fragments were treated with 1 μM dopamine and kept in culture for an additional 24 hours. On day 4, choroidal explants were dissociated with collagenase, and CD31^+^CD11b^–^ cells were sorted on a FACSMelody cell sorter (BD Biosciences). Cells were immediately flash-frozen in 96-well plates until further processing. RNA-Seq libraries were made from 500 sorted endothelial cells using SMART-Seq v4 Ultra Low Input RNA Kit (Takara Bio Inc.). RNA-Seq libraries were sequenced on a NovaseqX Illumina sequencer (iGENseq, ICM). FASTQ files were trimmed with Trimmomatic v0.39 using the NexteraPE-PE adapter file. The paired reads were then aligned using STAR (v2.7.9a) against the mouse reference genome from Ensembl v106 (generated June 21, 2022), with option “--quantMode GeneCounts.” All count files were concatenated into a single file, and a sample file including the conditions and the replicate numbers was generated. The count file and the sample file were loaded in EYE.DVseq, an in-house R Shiny application. 1 was added to all counts, and the genes having a total count less than 10 across all samples were removed. DESeq2 (v1.40.2) analysis was performed comparing the control and dopamine conditions. The results were then filtered for significant genes using the *P* values (<0.05).

### Cell proliferation assay.

Four thousand HCECs (36052-03, Celprogen) were seeded in a gelatin-coated 24-well plate, cultured for 24 hours in ECGM-2, serum-starved for 24 hours in Opti-MEM, and then treated with either PBS, dopamine, or l-DOPA, followed 10 minutes later by addition of 10 ng/mL VEGF. After culture for 24 hours, cells were fixed by 4% paraformaldehyde, and nuclei were stained with Hoechst and automatically counted under a fluorescent microscope (Arrayscan VTI HCS Reader, Thermo Fisher Scientific).

### Clinical cohort.

The French national health information database (Système National des Données de Santé [SNDS]) was used. This database collects all the reimbursement information for the entire French population in terms of medical and surgical procedures carried out, treatments provided, hospital admissions and their reasons, and long-term conditions giving entitlement to additional reimbursements. The study presented here is part of the French Epidemiology and Safety (EPISAFE) collaborative program (69). We designed an algorithm for identifying patients with nAMD, aged 50 years and older. Identification criteria were nAMD diagnosis, nAMD long-disease code, or reimbursement of nAMD treatments (anti-VEGF intravitreal injection, or dynamic phototherapy with verteporfin). Exclusion criteria were high myopia, diagnosis of other retinal diseases, treatments for other macular diseases (dexamethasone implant, retinal photocoagulation, etc.), or other retinal conditions. Included patients with nAMD needed to have had a minimum of 2 years of follow-up after their first IVT. The data extracted were age at first anti-VEGF IVT; sex; diabetes, chronic alcoholism, smoking, obesity, hypertension, and dyslipidemia status; number of IVTs performed in the first year, in the second year, and over the first 2 years; therapeutic class(es) of anti-parkinsonian treatment used; and anti-emetics deliveries. The daily dose of anti-parkinsonian treatment was approximated by the treatment packaging, the number of milligrams per presentation, and the frequency of dispensing. Naive patients with nAMD were defined as those naive of any IVT anti-parkinsonian treatment before first IVT. Comparisons were made using the χ^2^ test for categorical variables and the signed-rank test for continuous variables. Generalized linear models were computed. Statistical significance was set at *P* less than 0.05 (2-tailed tests). All data processing and statistical analyses were performed using the SAS statistical analysis software package (SAS Enterprise Guide version 7.1, SAS Institute Inc.).

### Statistics.

For pharmacoepidemiological data, the statistical methods are described above. For in vivo and in vitro experiments, GraphPad 7 (GraphPad Software) was used for data analysis. All values are reported in the scatter dot blots as well as the mean ± SEM. Statistical analysis and variance analysis were performed by 1-way ANOVA, corrected by Bonferroni’s post-test for multiple comparison, or Mann-Whitney *U* test for 2-group comparison depending on the experimental design.

### Study approval.

All animal experimental protocols and procedures adhered to the Association for Research in Vision and Ophthalmology Statement for the Use of Animals in Ophthalmic and Vision Research and were approved by the French Ministry of Higher Education, Research and Innovation (APAFIS authorization 18912 2019011417393998).

The clinical study was approved by the French Institute of Health Data (registration 115306, January 24, 2019) and by the French Data Protection Authority (registration D.R. 2019-100, April 12, 2019). This study adhered to the tenets of the Declaration of Helsinki.

### Data availability.

Experimental materials are available upon request with no restrictions. The RNA-Seq raw FASTQ files and count files were deposited in the NCBI’s Gene Expression Omnibus database (GEO GSE266525). The database used in the clinical study was transmitted by the French National Health Insurance Fund (Caisse nationale de l’assurance maladie [CNAM]), which is responsible for the extraction of SNDS data. The use of these data by our department was approved by the National Committee for Data Protection. We are not authorized to transmit these data. Data are available for researchers who meet the criteria for access to these French data from the CNAM: training for personal accreditation, and approval of the protocol by required authorities (Ethical and Scientific Committee for Research, Studies and Evaluations in the Health Field [CESREES] and National Committee for Data Protection [CNIL]). A Supporting Data Values file is available online as supplemental material.

## Author contributions

TM, CCG, CQ, SH, and FS conceptualized the study. TM, CCG, CQ, SH, MMF, and FS curated data. CCG, CQ, SH, JAS, MMF, LK, and FS acquired funding. TM, FB, ASM, SA, MB, LP, CR, ÉB, GB, CN, CCG, CQ, CD, XG, SH, MMF, LK, and FS collected data. CD, FB, ASM, ÉB, CQ, SH, and FS devised methodology. RP, CZ, JAS, CCG, CQ, SH, LK, and FS provided resources. CD, XG, CCG, CQ, SH, and FS supervised the study. TM, CCG, CQ, SH, and FS wrote the original draft of the manuscript. TM, FB, SH, and FS reviewed and edited the manuscript.

## Supplementary Material

Supporting data values

## Figures and Tables

**Table 1:**
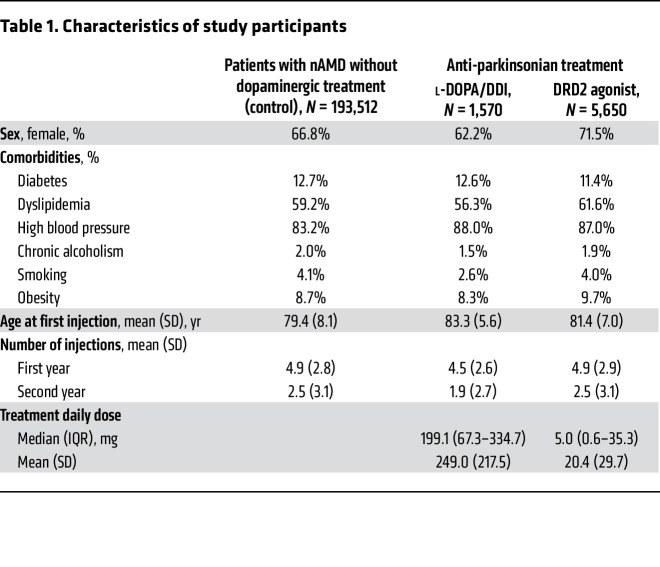
Characteristics of study participants

## Version 1 07/16/2024

In-Press Preview

## Version 2 09/03/2024

Electronic publication
